# Caffeine Abolishes the Ultraviolet-Induced REV3 Translesion Replication Pathway in Mouse Cells

**DOI:** 10.3390/ijms12128513

**Published:** 2011-11-29

**Authors:** Jun Takezawa, Naomi Aiba, Kagemasa Kajiwara, Kouichi Yamada

**Affiliations:** 1 Division of Genetic Biochemistry, The National Institute of Health and Nutrition, Shinjuku-ku, Tokyo 162-8636, Japan; E-Mail: junt@nih.go.jp (J.T.); 2 School of Medicine, Tokai University, Isehara-shi, Kanagawa-ken 259-1193, Japan

**Keywords:** alkaline sucrose density gradient sedimentation, caffeine, DNA polymerase ζ, proteasome inhibitor, TLS, translesion replication, *Rev3* knockout

## Abstract

When a replicative DNA polymerase stalls upon encountering a photoproduct on the template strand, it is relieved by other low-processivity polymerase(s), which insert nucleotide(s) opposite the lesion. Using an alkaline sucrose density gradient sedimentation technique, we previously classified this process termed UV-induced translesion replication (UV-TLS) into two types. In human cancer cells or xeroderma pigmentosum variant (XP-V) cells, UV-TLS was inhibited by caffeine or proteasome inhibitors. However, in normal human cells, the process was insensitive to these reagents. Reportedly, in yeast or mammalian cells, REV3 protein (a catalytic subunit of DNA polymerase ζ) is predominantly involved in the former type of TLS. Here, we studied UV-TLS in fibroblasts derived from the *Rev3*-knockout mouse embryo (*Rev3*KO-MEF). In the wild-type MEF, UV-TLS was slow (similar to that of human cancer cells or XP-V cells), and was abolished by caffeine or MG-262. In 2 cell lines of *Rev3*KO-MEF (*Rev3*^−/−^ *p53*^−/−^), UV-TLS was not observed. In *p53*KO-MEF, which is a strict control for *Rev3*KO-MEF, the UV-TLS response was similar to that of the wild-type. Introduction of the *Rev3* expression plasmid into *Rev3*KO-MEF restored the UV-TLS response in selected stable transformants. In some transformants, viability to UV was the same as that in the wild-type, and the death rate was increased by caffeine. Our findings indicate that REV3 is predominantly involved in UV-TLS in mouse cells, and that the REV3 translesion pathway is suppressed by caffeine or proteasome inhibitors.

## 1. Introduction

At least five mammalian DNA polymerases are suggested to be implicated in UV-induced translesion replication (TLS) (reviewed in [1]): Pols η, ι, ζ, κ and REV1. Polζ belongs to the B family, while the others belong to the Y family (reviewed in [1–3]). In particular, Polη and Polζ are markedly involved in UV-TLS in human [4] or vertebrate cells [5].

Polζ is a complex of the *Rev3* and *Rev7* gene products, which act as the catalytic and regulatory subunits, respectively. *Rev1*, *3*, and *7* were originally cloned from *Saccharomyces cerevisiae* isolates showing reduced frequency of UV-induced reversion of *cyc1* mutations [6]. Human and mouse homologs (*Rev1*, *3*, and *7*) were subsequently isolated [7–9]. Yeast Polζ is known to be responsible for 98% of UV-induced base substitutions and 90% of frameshift mutations, and also for spontaneous mutations [10]. Nonetheless, yeast Polζ was revealed to be too faithful to incorporate nucleotides opposite CPD. Instead, it can efficiently extend from a matched or mismatched 3′-end (reviewed in [2,3]). Human or mouse Polζ is assumed to have similar enzymatic properties to that of yeast; several lines of antisense RNA expression or siRNA knockdown experiments in human or mouse cells have shown that Polζ is involved in mutagenic TLS [11–13].

Polη was first purified from a HeLa cell extract as an activity that complements TLS defect in a xeroderma pigmentosum variant (XP-V) cell extract [14]. Patients with the autosomal recessive disorder, XP-V, have a predisposition to skin cancer. Following UV irradiation, XP-V cells demonstrate hypermutability (reviewed in [15]). Thus, Polη is the product of a defective gene in XP-V. Human Polη was also identified via a homolog search of the yeast *S. cerevisiae Rad30* gene, which encodes an error-free bypass protein [16]. Various XP-V causative mutations have been found in the *Polη* gene, *hRAD30A*, of XP-V patients [16,17].

In the lesion-bypass assay, human Polη was shown to catalyse DNA synthesis past TT-CPD efficiently and in a relatively accurate manner [3,16,18]. When template DNA contained a (6–4)TT-PP, Polη incorporated 1 (random) nucleotide opposite the first thymine, and another nucleotide opposite the second thymine of the lesion, but rarely elongated beyond this point [3,14,18].

Intrinsically, both yeast and human Polη incorporate the wrong nucleotide at a fairly high rate. However, they can extend these mismatched primer termini with only a frequency of ~10^−2^ to 10^−3^ relative to extension from the matched primer termini [3,19]. Plausibly, Polη dissociates from there and the proofreading exonuclease of Polδ removes the wrong nucleotide [20]. We suppose that disruption or malfunction of this cooperation renders mismatched primer termini accessible to Polζ.

As described above, UV-TLS by Polη is thought to be relatively accurate *in vivo*. Recently, however, a more complicated contribution of multiple bypass polymerases to TLS was documented [21–24].

To detect TLS, an alkaline sucrose density gradient centrifugation (ASDG) technique is typically used. Pulse-labeled replication products are smaller in UV-irradiated XP-V cells than in unirradiated cells; however, on prolonged incubation, the replication products in the irradiated cells eventually attain a high molecular weight, similar to that in unirradiated cells. This conversion is interpreted that DNA synthesis is temporarily retarded by UV photoproducts, and then continues beyond the lesion, leaving a gap that is subsequently sealed [25]. The initial size of the newly synthesized DNA is approximately equal to the average distance between lesions in the template strands [26]. This suggests that the gaps in the nascent DNA are opposite the photoproducts [27]. Therefore, sealing of the gaps, by translesion or other postreplication repair mechanisms, can be observed by monitoring the molecular weight of labeled DNA.

Using a modified ASDG technique [28], we previously detected the conversion of pulse-labeled replication products in UV-irradiated fibroblasts derived from XP-V patients, showing that UV-TLS is delayed in the cells, but not completely abolished [29]. The marginal TLS was markedly prevented by caffeine at millimolar concentrations [25,29], and also by proteasome inhibitors (unpublished results). By contrast, these reagents did not retard UV-TLS in normal diploid cells.

To further investigate the inefficient polymerase(s) which may be sensitive to caffeine and proteasome inhibitors *in vivo*, we added specific DNA polymerase inhibitors. Butylphenyldeoxy-guanosine (BuPGdR) inhibited TLS in XP-V cells [29], suggesting that Polζ may be involved in this process.

We further revealed that these reagents also inhibited UV-TLS in human cancer cells [30]. Moreover, similar to XP-V cells, UV-TLS was much slower in human cancer cells than in normal human cells. These findings indicate that UV-TLS in cancer cells is predominantly of the Polζ-dependent type.

Using siRNAs, we recently verified the participation of multiple bypass polymerases in UV-TLS in HeLa cells [6]: Polζ plays a primary role, while REV1 is indispensable in mutagenic TLS. Unexpectedly, siRNAs to Polη prevented TLS to a large extent, implying that the Polη- and Polζ-dependent processes are not alternatives but overlap. Polκ, but not Polι, participates in this UV-TLS partially.

In the present study, to obtain more definite results about the target(s) of these reagents, we studied UV-TLS in fibroblasts derived from the *Rev3*-knockout mouse embryo (*Rev*3KO-MEF), previously constructed by one of the authors (K.K.). The *Sez4* gene was originally isolated from the mouse brain following a pentylenetetrazol-induced seizure, and found to be a mouse homolog of *S. cerevisiae Rev3* [31]. (This was a partial sequence, but the first publication of the mouse mRNA sequence.) To study the role of Polζ in seizure, we inactivated the *Rev3* gene in embryonic stem (ES) cells, injected them into blastocysts, established *Rev3*^+/−^ mice, and intercrossed. The resultant *Rev3*^−/−^ mice died around embryonic day (E) 10.5 [32]. Neither the resulting MEF nor the *Rev3*^−/−^ ES cells could be propagated. To rescue the embryonic lethality in midgestation, we introduced a *Rev3* transgene, or inactivated *p53* gene. These attempts were unsuccessful, although the embryos survived until E12.5–E13.5 [32] or E12.5 [33], respectively. However, *Rev3*-Tg^+^ *Rev3*^−/−^ blastocysts could be proliferated *in vitro* [33]. Then, we successfully constructed *Rev3*^−/−^ *p53*^−/−^ MEFs and *Rev3*-Tg^+^ *Rev3*^−/−^ MEFs from the embryos.

Our results concerning *Rev3* gene targeting generally agree with those of other researchers (reviewed in [34,35]). Polζ is essential for cell viability during embryonic development [32,36–39], probably because TLS ability of Polζ is indispensable in tightly scheduled replication and expression of embryogenic genes. At least 4 groups (including ours) generated *Rev3*^−/−^ *p53*^−/−^ MEFs [40–42] and studied the properties of Polζ. MEFs were shown to be hypersensitive to UV [40,41] or cisplatin [41], and to accumulate in the S and G_2_/M phases following these damages [41]. Other studies demonstrated that MEFs were moderately sensitive to UV, γ-radiation, methyl methanesulfonate, or mitomycin C [42]. A significant increase in double-strand DNA breaks [39], chromosomal instability (chromosome and chromatid breaks, chromatid exchanges, and translocations) [39,42], increase in chromosomal numbers, and double-minute chromosomes [42] were observed.

Using ASDG, Jansen *et al*. reported defect in postreplication repair in UV-irradiated *Rev3*^−/−^ *p53*^−/−^ MEFs [40]. Besides, unlike in *Rev1*-deficient MEFs, there was no delay in fork progression after UV irradiation (results from DNA combing assay). While 1 h after UV irradiation, the progression stopped completely in the *Rev3*^−/−^ *p53*^−/−^ MEFs (results from alkaline DNA-unwinding assay), inferring that *Rev3* is essential for a late mode of DNA damage tolerance (post-replicative gap filling).

In the present study, we investigated the effects of caffeine and proteasome inhibitors on UV-TLS, and also the kinetics of progression for the nascent strand in UV-irradiated *Rev3*^−/−^ *p53*^−/−^ MEFs.

## 2. Results and Discussion

### 2.1. Detection of UV-Induced Translesion Replication in Mouse Cells

**Figure 1:**
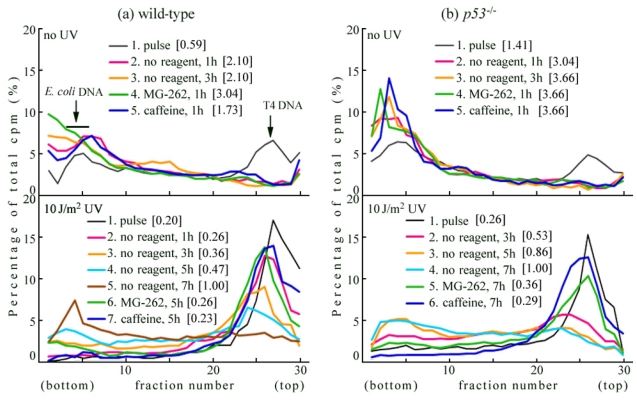
Alkaline sucrose density gradient centrifugation (ASDG) profiles of replication products in (**a**) wild-type and (**b**) *p53*^−/−^ mouse embryo fibroblasts (MEFs). (Time course and effects of MG-262 or caffeine); (**upper panel**) Non-irradiated cells were pulse-labeled with 10 μCi/mL of [^14^C]-thymidine for 30 min, and chased in normal medium for 1 h or 3 h. Similarly, pulse-labeled cells were chased for 1 h in normal medium containing 5.0 μM MG-262 or 5 mM caffeine; (**lower panel**) Cells were irradiated with 10 J/m^2^ UV and incubated for 30 min. The cells were then pulse-labeled with 10 μCi/mL of [^14^C]-thymidine for 1 h, and chased in normal medium for 1 h, 3 h, 5 h, or 7 h. Similarly, pulse-labeled cells were chased for 5 h or 7 h in normal medium containing 5.0 μM MG-262 or 5 mM caffeine, respectively. Sedimentation is from right to left. The arrow indicates the position of T4 phage DNA (166 kb, *i.e.*, approximately 5.5 × 10^7^ Da/single strand). Labeled *E. coli* DNA (approximately 4 Mb) sedimented near the bottom (fractions 3–6) [28]. The average fragment length (in Mb) of each profile is shown in square brackets. cpm: counts per minute.

Initially, we studied UV-TLS in wild-type MEFs (lower panel of Figure 1a). Wild-type cells were irradiated with UV (10 J/m^2^) and incubated for 30 min. The cells were then pulse-labeled with [^14^C]-thymidine for 1 h. The replication products immediately after UV irradiation were sedimented as a sharp peak (illustrated by a thin black line in the panel—line 1) similar in size to that of the T4 phage DNA marker. When the cells were chased in the normal medium for 1 h (line 2), 3 h (line 3), 5 h (line 4), or 7 h (line 5), the products gradually joined to form larger DNA, with lengths in the order of megabases (Mb). The conversion (*i.e.*, joining of the fragments into chromosomal size, indicating the TLS reaction) was very slow compared to that of human normal cells such as NB1RGB [29,30]. This slow conversion was also observed in XP-V cells [29] and human cancer cells such as HeLa cells [30].

Wild-type cells were pulse-labeled as described above in the absence of reagent, and chased for 5 h in a medium containing 5 μM MG-262 or 5 mM caffeine. MG-262 and caffeine significantly delayed the conversion in UV-irradiated wild-type cells (lower panel of Figure 1a—compare lines 6 and 7 with line 4), indicating that the UV-TLS process in these mouse cells was sensitive to proteasome inhibitors or caffeine. By contrast, in the no-UV controls (*i.e.*, normal replication), these inhibitors had no effect (upper panel of Figure 1a—compare lines 4 and 5 with line 2).

In the *p53*^−/−^ cells (which are strict controls for *Rev3*^−/−^*p53*^−/−^), the conversion (*i.e.*, joining of the fragments into chromosomal size) was also very slow (lower panel of Figure 1b—lines 2, 3, 4), and UV-TLS was delayed by MG-262 and caffeine (—compare lines 5 and 6 with line 4). In the *p53*^−/−^ cells, replication proceeded without delay (upper panel of Figure 1b—lines 2 and 3). Reportedly, after UV irradiation, p53 stimulated monoubiquitination of proliferating cell nuclear antigen (PCNA) via p21, and up-regulated TLS [43]. It was also reported that UV-induced downregulation of *p21* caused efficient PCNA ubiquitination [44]. However, our present results indicate that the levels of UV-TLS are not affected by p53, and that inhibition by MG-262 and caffeine is unaffected, even in the *p53*^−/−^ cells. We also detected UV-TLS in human cancer strains, irrespective of p53 status [30].

### 2.2. UV-TLS Was Mostly Abolished in Rev3 Knockout MEFs

**Figure 2:**
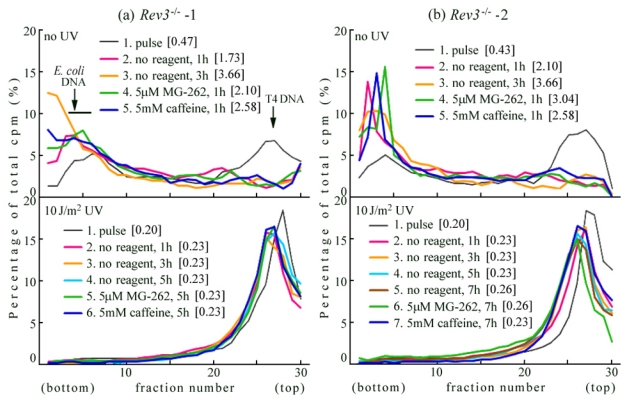
ASDG profiles of replication products in (**a**) *Rev3*^−/−^-1 and (**b**) *Rev3*^−/−^-2 MEFs. (Time course and effects of MG-262 or caffeine). (**upper panel**) Non-irradiated cells were pulse-labeled with 10 μCi/mL of [^14^C]-thymidine for 30 min, and chased in normal medium for 1 h or 3 h. Similarly, pulse-labeled cells were chased for 1 h in normal medium containing 5.0 μM MG-262 or 5 mM caffeine; (**lower panel**) Cells were irradiated with 10 J/m^2^ UV and incubated for 30 min. These cells were pulse-labeled with 10 μCi/mL of [^14^C]-thymidine for 1 h, and chased in normal medium for 1 h, 3 h, 5 h, or 7 h. Similarly, pulse-labeled cells were chased for 5 h or 7 h in normal medium containing 5.0 μM MG-262 or 5 mM caffeine, respectively. Sedimentation is from right to left. The arrow indicates the position of T4 phage DNA (166 kb, *i.e.*, approximately 5.5 × 10^7^ Da/single strand). Labeled *E. coli* DNA (approximately 4 Mb) sedimented near the bottom (fractions 3–6) [28]. The average fragment length (in Mb) of each profile is shown in square brackets. cpm: counts per minute.

Two cell lines of *Rev3*^−/−^ *p53*^−/−^ were primary cultured from the E9.5 embryo. In the *Rev3*^−/−^-1 cells, the replication products immediately after UV irradiation were sedimented as a sharp peak, which was slightly smaller than that of the marker T4 phage DNA (lower panel of Figure 2a—line 1 [0.20 Mb]). When the cells were chased in the normal medium for 1 h, the products joined to form slightly larger DNA (illustrated by a red line—line 2). However, on further incubation, the DNA fragments remained small ([0.23 Mb]—compare lines 3 and 4 with line 2). These results were reproducible, not only in the *Rev3*^−/−^-1 cells, but also in the *Rev3*^−/−^-2 cells (lower panel of Figure 2b). Above results indicated that polζ is required for UV-TLS in mouse cells.

When these *Rev3* null cells were pulse-labeled in the absence of reagents, and chased for 5 h in a medium containing 5 μM MG-262 or 5 mM caffeine, the profiles coincided with that of the cells chased in the normal medium for 1 h (lower panel of Figure 2a—compare lines 5 and 6 with line 2). Similar results were obtained in the *Rev3*^−/−^-2 cells. It is likely that bypass polymerase(s) other than Polζ, with no sensitivity to MG-262 or caffeine, contribute to this minor conversion (*i.e.*, the slight change from [0.20 Mb] to [0.23 Mb]).

We observed no delay in normal replication (upper panel of Figure 2a,b—lines 2 and 3), suggesting that replication proceeds in the absence of Polζ.

### 2.3. Rev3 Transgene Restored UV-TLS in Rev3 Knockout Mouse Cells

**Figure 3:**
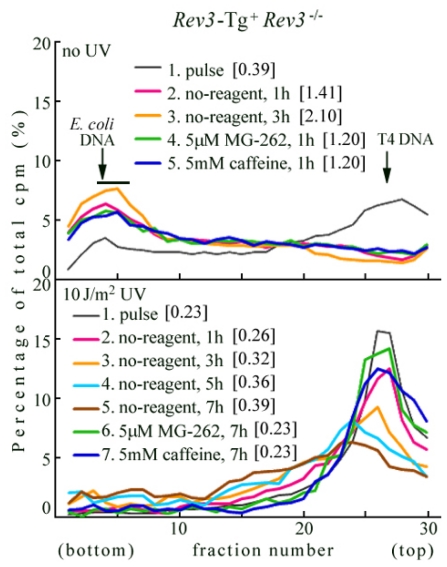
ASDG profiles of replication products in *Rev3*-Tg^+^ *Rev3*^−/−^ MEFs. (Time course and effects of MG-262 or caffeine). (**upper panel**) Non-irradiated cells were pulse-labeled with 10 μCi/mL of [^14^C]-thymidine for 30 min, and chased in normal medium for 1 h or 3 h. Similarly, pulse-labeled cells were chased for 1 h in normal medium containing 5.0 μM MG-262 or 5 mM caffeine. (**lower panel**) Cells were irradiated with 10 J/m^2^ UV and incubated for 30 min. The cells were then pulse-labeled with 10 μCi/mL of [^14^C]-thymidine for 1 h, and chased in normal medium for 1 h, 3 h, 5 h, or 7 h. Similarly, pulse-labeled cells were chased for 7 h in normal medium containing 5.0 μM MG-262 or 5 mM caffeine. Sedimentation is from right to left. The arrow indicates the position of T4 phage DNA (166 kb, *i.e.*, approximately 5.5 × 10^7^ Da/single strand). Labeled *E. coli* DNA (approximately 4 Mb) sedimented near the bottom (fractions 3–6) [28]. The average fragment length (in Mb) of each profile is shown in square brackets. cpm: counts per minute.

*Rev3*-Tg^+^ *Rev3*^−/−^ MEFs were primary cultured from the E10.5 embryo selected from cohort intercrossed *Rev3* transgenic mice with *Rev3*^+/−^ mice [33]. In *Rev3*-Tg^+^ *Rev3*^−/−^ MEFs (lower panel of Figure 3), the replication products immediately after UV irradiation were sedimented as a sharp peak (line 1). When the cells were chased in normal medium for 1 h (line 2), 3 h (line 3), 5 h (line 4), or 7 h (line 5), the products gradually joined to form larger DNA. The conversion was considerably slower than in wild-type MEFs (lower panel of Figure 1a). Our results suggest that the *Rev3* transgene partially restores *Rev3* expression in *Rev3*KO-mice. This may explain why the *Rev3* transgene previously only partially restored normal embryogenesis in mice [33].

In *Rev3*-Tg^+^ *Rev3*^−/−^ MEFs, we also observed that conversion was retarded by MG-262 or caffeine (lower panel of Figure 3—compare lines 6 and 7 with line 5). This suggests that the UV-TLS response restored by the *Rev3* transgene is sensitive to proteasome inhibitors and caffeine.

### 2.4. Introduction of the Rev3 Expression Vector Restored UV-TLS in Rev3 Knockout MEFs

**Figure 4:**
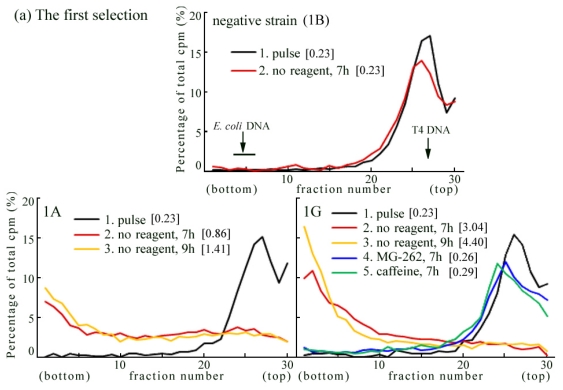

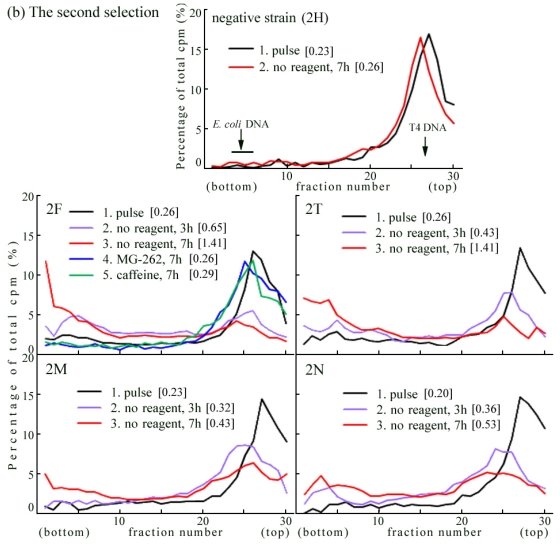
Introduction of the mouse *Rev3* expression vector into *Rev3*^−/−^-1 MEFs. (**a**) the first selection and (**b**) the second selection. (Time course and effects of MG-262 or caffeine by ASDG) The vector containing mouse *Rev3* cDNA was introduced into *Rev3*^−/−^-1 cells. In the first selection, the blasticidine *S*-resistant cell lines were picked up, and their UV-TLS responses were observed by ASDG. In the second selection, the plasmid was linearized by *Fsp*I digestion and transfected into *Rev3*^−/−^-1 cells. Sedimentation is from right to left. The arrow indicates the position of T4 phage DNA (166 kb, *i.e.*, approximately 5.5 × 10^7^ Da/single strand). Labeled *E. coli* DNA (approximately 4 Mb) sedimented near the bottom (fractions 3–6) [28]. The average fragment length (in Mb) of each profile is shown in square brackets. cpm: counts per minute.

We transfected the pcDNA6 recombinant plasmid, containing full-length mouse *Rev3* cDNA, into *Rev3*^−/−^-1 cells (*i.e.*, *Rev3*^−/−^ *p53*^−/−^). We initially picked up 16 blasticidine *S*-resistant strains, and observed conversion of their replication product following UV irradiation by ASDG. We detected UV-TLS in 2 of the 16 cell lines (1A and 1G) (Figure 4a).

Next, the pcDNA6 recombinant plasmid was linearized by *Fsp*I digestion and transfected into *Rev3*^−/−^-1 cells. We picked up 18 blasticidine *S*-resistant strains, and detected complete conversion of UV-TLS (*i.e.*, the same level of conversion as in wild-type MEFs) in 2 of these strains (2F and 2T) (Figure 4b). In 6 strains (*e.g.*, 2M, 2N), we observed partial UV-TLS, while in 4 strains we observed only slight UV-TLS (*i.e.*, very slow conversion conversion) (data not shown).

The addition of 5 μM MG-262 or 5 mM caffeine to the chase medium significantly delayed conversion in UV-irradiated 1G or 2F cells (Figure 4: 1G and 2F—compare lines 4 and 5 with line 2 or line 3). These results indicate that the expression of exogenous *Rev3* in *Rev3*KO-MEFs restores the UV-TLS response, and that this response is sensitive to proteasome inhibitors and caffeine.

### 2.5. Correlation of UV Survival with UV-TLS

**Figure 5:**
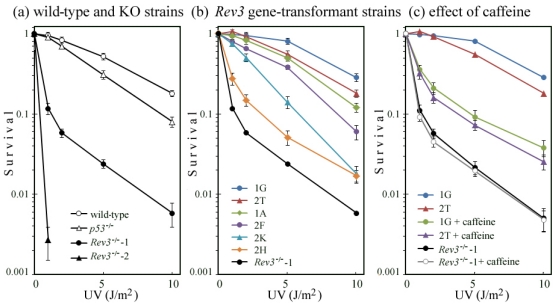
UV survival of: (**a**) wild-type and knockout (KO) strains; and (**b**) *Rev3* gene-transformed strains. Cells were cultured in normal medium without caffeine; (**c**) 1.0 mM caffeine was added to the culture medium, until the surviving cells had formed colonies (1G, 2T and *Rev3*^−/−^-1). UV survival of cells was measured by colony formation. Data are presented as mean survival rates (SD).

UV survival of cells was measured by colony formation in the absence of caffeine or MG-262. We observed that *p53*^−/−^ MEFs were more sensitive to UV than were wild-type MEFs. On the other hand, *Rev3*^−/−^-2 MEFs were more sensitive to UV than were *Rev3*^−/−^-1 MEFs (Figure 5a). The reason for this difference in UV sensitivity is unknown, because the 2 cell lines were isolated from the same embryo.

Next, we investigated the UV survival of *Rev3* gene-transformed cell lines derived from *Rev3*^−/−^-1 MEFs. We observed that UV survival of these cell lines was mostly in parallel with their abilities for TLS (Figure 5b). The survival curves for 1A, 1G, 2T, and 2F, in which UV-TLS was executed at a similar speed to that of the wild-type (Figure 4), were comparable to those of the wild-type or *p53*^−/−^ MEF. By contrast, 2K, in which UV-TLS was executed very slowly (data not shown), was more sensitive to UV than were the wild-type or *p53*^−/−^ MEF. Meanwhile, the survival curve of 2H, in which no UV-TLS was detected, closely resembled that of *Rev3*^−/−^-1 cells.

The addition of 1 mM caffeine to the medium decreased the survival of 1G and 2T to the level of 2H (Figure 5c). This suggests that caffeine abolishes the UV-induced REV3 TLS pathway, and causes death of mouse cells. By contrast, even in the presence of caffeine, *Rev3*^−/−^-1 MEFs were as sensitive as in the absence. Their survival curves are in excellent agreement (Figure 5c). These results suggest that, in UV-induced cell death, there are few targets for caffeine except for the REV3 pathway.

We were unable to determine the effects of proteasome inhibitor on UV survival, because of the cytotoxicity of MG-262.

### 2.6. Where Is the Target for UV-TLS Inhibition by Caffeine or Proteasome Inhibitors?

Caffeine has pleiotropic effects on DNA metabolism such as DNA synthesis, apoptosis, chromatin condensation, and also on the cell-cycle checkpoint [45]. Caffeine is a known inhibitor of c-AMP phosphodiesterase, ATM (ataxia-telangiectasia mutated) kinase or ATR (ataxia-telangiectasia and Rad*3*-related) kinase. However, involvement of ATM kinase in this UV-TLS is less probable, because flow cytometry analysis showed caffeine did not cause an “override” of *S*-arrest in 2 UV-irradiated XP-V cell lines [29].

To study relevance between augmentation of cAMP levels and caffeine-induced apoptosis after UV, Heffernan *et al*. [46] used a cell-permeable analog of cAMP and observed no caffeine-like effects in UVB-irradiated human keratinocytes. (UV-induced apoptosis is thought to mainly result from a failure in TLS.) Therefore, the target of caffeine is not likely to be cAMP phosphodiesterase.

**Figure 6:**
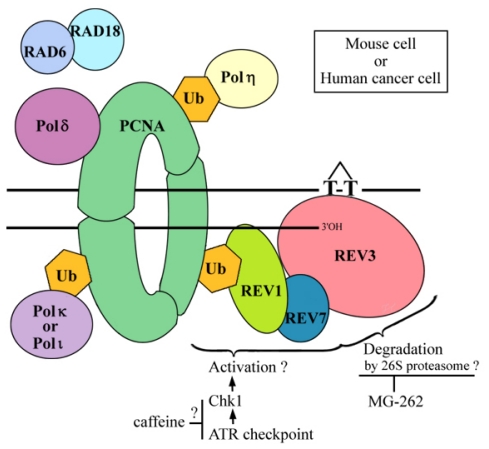
Possible targets for UV-TLS inhibition by proteasome inhibitors and caffeine.

Although the detailed mechanism of UV-TLS inhibition remains unexplained, the target for the inhibition must be a Ser/Thr kinase [47], which affects DNA damage tolerance. Upon UV irradiation, the ATR/Chk1 checkpoint pathway is activated. Using the DNA-combing method, Despras *et al*. [48] demonstrated that the pathway was over-activated in UV-irradiated XP-V cells, and that caffeine significantly decreased Chk1 activation and increased fork stalling. Furthermore, the Chk1-specific inhibitor, UCN-01, had an effect similar to caffeine [48], indicating that the UV-TLS target of caffeine may be ATR kinase. This hypothesis is compatible with the fact that caffeine does not prevent UV-TLS in normal cells, because the ATR/Chk1 pathway does not dominate monoubiquitination of PCNA [49] (which recruits Polη to the lesion site). In the present study, it has been shown that caffeine inhibits REV3-dependent UV-TLS in mouse cells. However, the phosphorylation target of ATR/Chk1 pathway has not been identified in this system (Figure 6).

We propose that the 26S proteasome is involved in UV-TLS in XP-V and cancer cells. Using epistatic analysis in *S. cerevisiae*, Podlaska and co-workers [50,51] demonstrated a link between 20S proteasomal activity and postreplication repair. Postreplication repair is the mechanism by which stalling of the replication fork is avoided and includes TLS and template switching. Both phenomena can be detected by pulse-chase experiment and ASDG. However, the likely target protein for signaling by polyubiquitins, and also degradation by the proteasome, has yet to be determined (Figure 6).

Since the 1970s, there has been controversy about the inhibitory effect of caffeine on postreplication repair. The following consensus has been reached [52]. In rodent cells, following UV irradiation, caffeine decreases the survival rate and mutation frequency, and inhibits postreplication repair. Moreover, it may enhance chromosomal aberrations in cell strains cultured from mice or Chinese hamsters. By contrast, in humans, the effects of caffeine vary among cell types: in normal fibroblasts, it has no effect on survival and postreplication repair; in XP-V cells, it decreases survival and inhibits postreplication repair; and in classical type XP cells, it has no effect on survival, and only a slight inhibitory effect on postreplication repair [53].

In this paper, we have clearly demonstrated that caffeine targets the REV3 TLS pathway [54]. Moreover, proteasome inhibitors target the same pathway. Our findings are compatible with the above consensus. Further studies are required to identify the TLS enzymes blocked by caffeine or proteasome inhibitors, and also to clarify the detailed mechanisms of inhibition.

## 3. Experimental Section

### 3.1. Mouse Cell Culture

Cell lines were primary cultured from mouse embryos and established previously by one of the authors (K.K.). Briefly, the mouse embryo was finely minced and treated with 0.05% trypsin-EDTA at 37 °C for 10 min. The detached cell suspension was cultured in a 1:1 mixture of Dulbecco’s modified Eagle’s medium (DMEM) with 4.5 g/L glucose, and Ham’s F12 supplemented with 1% nonessential amino acids, 1% glutamine, and 10% fetal calf serum (FCS). After 1 week of culture, the medium was changed into normal medium.

Mouse cells were maintained in monolayers in DMEM supplemented with 10% FCS (“normal” medium), trypsinized, and seeded into culture dishes (1 × 10^5^ cells/Φ 60-mm dish). Collagen-coated dishes (Collagen Type IV Cellware, BD Falcon) were used for wild-type and *Rev3*-Tg^+^ *Rev3*^−/−^ MEFs.

### 3.2. UV Irradiation and Translesion Replication

Mouse cells were exposed to UV light (10 J/m^2^) from a germicidal lamp (Toshiba GL15) at 0.6 J/m^2^ s. After 30 min of culture, the medium was changed to labeling medium consisting of DMEM supplemented with 10% FCS and 10 mCi/mL of [*U*-^14^C]-thymidine (Moravek MC267, 470 mCi/mmol). UV-irradiated cells were pulse-labeled for 1 h, while non-irradiated cells were labeled for 30 min. The medium was changed to normal medium with or without MG-262 (Wako) or caffeine (Sigma), and the cells were chased for 1 h, 3 h, 5 h, or 7 h. The dosages of MG-262 (5 μM) or caffeine (5 mM) were checked previously [29,30], and fixed throughout the experiments. The cells were harvested by trypsinization and examined by alkaline sucrose density gradient centrifugation (ASDG) [28].

### 3.3. Alkaline Sucrose Density Gradient Centrifugation

Cells (approximately 1 × 10^5^ in 50 μL of phosphate buffered saline [PBS]) were gently layered onto 50 μL of 1% sucrose in PBS, which was overlaid on 100 μL of lysis solution (0.6 M KOH, 2.0 M KCl, 10 mM EDTA, and 1% *N*-lauroylsarcosine), which was placed on top of a 4.35-mL alkaline 5–20% sucrose gradient (0.3 M KOH, 2.0 M KCl, 1 mM EDTA, and 0.1% *N*-lauroylsarcosine) with 0.4 mL of alkaline 80% sucrose as a cushion at the bottom. The gradient was centrifuged at 6000 rpm (4320 × *g*) for 15.6 h at 15 °C in a Beckman SW 50.1 rotor. The gradient was fractionated onto 30 circles of No. 17 paper (Whatman). The paper circles were dried, immersed in cold 5% trichloroacetic acid for 10 min, washed 3 times with ethanol and once with acetone, and dried; radioactivity was then measured. As a molecular-weight marker, [^14^C]-labeled T4 DNA phage particles were placed on the lysis layer and sedimented in a parallel run. The approximate fragment length of each fraction was estimated on the basis of the positions of the T4 DNA marker and *E. coli* DNA, and adjusted by sucrose density curve [28]. The average fragment length (in megabases [Mb]) of each profile is shown in Figures 1–4 as the fragment length of the median fraction [29]. (The median fraction is the middle fraction that separates the higher half of the profile from the lower half.) These experiments were performed at least three times except in Figure 4 (twice).

### 3.4. Construction of the Mouse Rev3-Expressing Plasmid

The mouse *Rev3*-expressing plasmid was constructed by one of the authors (K.K.). A full-length mouse *Rev3* cDNA was assembled by reverse transcription polymerase chain reaction (RT-PCR) from pentylenetetrazol-treated primary cultured mouse neurons. The cDNA was initially isolated as a seizure-related gene, *Sez4* (NCBI accession no: AB031049). The full-length mouse *Rev3* cDNA was subcloned into the pCAGGS vector and introduced into mice for transgene [32,33]. The *Eco*RI fragment of the pCAGGS recombinant, containing mouse *Rev3* cDNA sequence (1st ATG: atgttttct; to *C*-terminal: ttactggag), was then recombined into the multicloning site of pBluescript (Fermentas). Next, the *Kpn*I*-Not*I fragment of the plasmid containing the entire mouse *Rev3* cDNA sequence was recombined into the pcDNA6/V5-His(C) vector (Invitrogen). (The full-length mouse *Rev3* cDNA was inserted between CMV promoter and V5 epitope of the vector.) The sequence of the resultant plasmid was confirmed.

### 3.5. Transfection with the Plasmid and Selection of Stable Transformant Strains

*Rev3*^−/−^-1 cells were seeded into culture dishes (2.5 × 10^5^ cells/Φ 100-mm dish). The following day, the cells were transfected with FuGENE6 (Roche)–plasmid DNA complex. After 2 days of culture, 4 μg/mL of blasticidine S (Invitrogen) was added to the medium. After 1 or 2 days of culture, cells were trypsinized, diluted and replated onto Φ 100-mm dishes. The cells were cultured for 7–12 days in the presence of 5 μg/mL blasticidine. Blasticidine-resistant colonies were picked up and propagated.

### 3.6. Survival Assay of UV-Irradiated Cells

Cell survival was measured by colony formation. Mouse cells were seeded into culture dishes at cloning densities. The following day, the cells were exposed to various doses of UV light, as described above. The cells were cultured in normal medium with or without 1.0 mM caffeine, until the surviving cells had formed colonies (approximately 14 days after UV exposure). The cells were then fixed and stained, and the colonies were counted. These experiments were performed in duplicate at least three times.

## 4. Conclusions

Using the ASDG technique, we studied UV-TLS in mouse cells. In the wild-type MEF, the UV-TLS response was slow (similar to that of human cancer cells or XP-V cells), and was abolished by caffeine or MG-262. In 2 cell lines of *Rev3*KO-MEFs (*Rev3*^−/−^ *p53*^−/−^), UV-TLS was not observed. In *p53*KO-MEF, which is a strict control for *Rev3*KO-MEF, the UV-TLS response was similar to that of the wild-type. These results indicate the participation of the *Rev3* gene product (a catalytic subunit of Polζ) in the UV-TLS pathway of mouse cells. Introduction of the *Rev3* expression plasmid into *Rev3*KO-MEF restored the UV-TLS response, which is sensitive to caffeine or MG-262, in selected stable transformants. Furthermore, in these transformants, viability to UV was the same as that in the wild-type, and caffeine causes death of mouse cells. In *Rev3*-Tg^+^ *Rev3*^−/−^ MEFs, UV-TLS was also restored by the *Rev3* transgene, and was inhibited by caffeine or MG-262. Taken together, our findings indicate that Polζ predominantly executes UV-TLS, not only in human cancer cells or XP-V cells, but also in mouse cells. Moreover, caffeine or proteasome inhibitors target the REV3 TLS pathway.
